# Improving treatment for patients with childhood abuse related posttraumatic stress disorder (IMPACT study): protocol for a multicenter randomized trial comparing prolonged exposure with intensified prolonged exposure and phase-based treatment

**DOI:** 10.1186/s12888-018-1967-5

**Published:** 2018-12-12

**Authors:** D. A. C. Oprel, C. M. Hoeboer, M. Schoorl, R. A. De Kleine, I. G. Wigard, M. Cloitre, A. Van Minnen, W. Van der Does

**Affiliations:** 1 Leiden University, Institute of Psychology, Wassenaarsweg 22, 3332 AK Leiden, The Netherlands; 2 Parnassiagroep, PsyQ, Lijnbaan 4, 2512 VA The Hague, The Netherlands; 3 Department of Clinical Psychology, University of Amsterdam, Overschiestraat 61, 1062 XD Amsterdam, The Netherlands; 4 Department of Psychiatry and Behavioral Sciences, Stanford University, Palo Alto, CA USA; 5 National Center for PTSD Dissemination and Training Division, Palo Alto Veterans Affairs Palo Alto Health Care System, 795 Willow Road, Menlo Park, CA USA; 6 PSYTREC, Prof. dr. Bronkhorststraat 2, 3723 MB Bilthoven, The Netherlands; 7 Radboud University, Behavioural Science Institute, Nijmegen, The Netherlands; 8 Department of Psychiatry, Leiden University Medical Center, Leiden, The Netherlands

**Keywords:** Posttraumatic stress disorder, CA-PTSD, Trauma focused treatment, Childhood trauma, Prolonged exposure, Phase-based treatment, Intensive treatment, STAIR

## Abstract

**Background:**

Childhood abuse related posttraumatic stress disorder (CA-PTSD) is associated with a high burden of disease and with treatment response rates that leave room for improvement. One of the treatments for PTSD, prolonged exposure (PE), is effective but has high drop-out rates and remission rates are relatively low. An intensified form of PE (iPE) was associated with good response and low drop-out rates in PTSD and has not yet been tested in a controlled trial in CA-PTSD. Phase-based treatment (PBT), in which PE is preceded by skills training may improve overall outcomes in this population. We will assess the effectiveness and cost-effectiveness of standard PE, iPE and PBT in patients with CA-PTSD.

**Methods/design:**

Multi-center randomized controlled trial. Treatment conditions are: prolonged exposure (PE; maximum of 16 sessions in 16 weeks); intensified PE (iPE; maximum of 12 sessions in four weeks and two booster sessions); phase-based treatment (PBT; maximum of eight sessions skills training followed by eight sessions PE in 16 weeks).

Primary outcome: Clinician-rated PTSD symptom severity. Secondary outcomes: loss of PTSD diagnosis, self-reported PTSD symptom severity, comorbid symptom severity and quality of life. Moreover, we will examine cost-effectiveness and moderators and mediators of treatment outcome. Target population: adults with CA-PTSD (*N* = 150). Assessments in weeks 0, 4, 8, 16, 26 and 52.

**Discussion:**

Given that no consensus yet exists about the treatment guidelines for patients with CA-PTSD, the present study may have important implications for the treatment of CA-PTSD.

**Trail registration:**

Registered at C.C.M.O. on Sept 7, 2016 (NL57984.058.16); retrospectively registered at June 21, 2017 at clinicaltrials.gov identifier: NCT03194113.

## Background

Childhood abuse is associated with severe negative long-term consequences. These include health problems, high health care utilization, a high risk of revictimization, low socio-economic well-being and criminal behavior in adulthood [1–6]. Childhood abuse is also related to many mental health problems such as depression, suicidality, dissociation, personality disorders, substance abuse and aggression [4, 5, 7–10]. In many cases, childhood abuse leads to Posttraumatic Stress Disorder (PTSD): 22 to 49% of those who report childhood abuse fulfill criteria for lifetime PTSD [11]. The treatment of PTSD in this population is relatively under investigated.

In international guidelines of PTSD, trauma-focused treatment (TFT) is recommended as first treatment option [12]. Substantial evidence exists for the effectiveness of TFT in patients with PTSD [13–15]. Treatment adherence and efficacy are relatively low, however. A meta-analysis indicated that 44% of the patients still fulfilled diagnostic criteria for PTSD at the end of treatment [14]. TFT may be less effective in CA-PTSD than in PTSD in general, because patients with CA-PTSD have more comorbid symptoms, such as interpersonal problems and emotion regulation difficulties [16]. These symptoms contribute significantly to functional impairment [17] but are not specifically addressed in TFT. This may lead to poorer outcomes and specifically less effective use of trauma focused interventions. The current study is designed to investigate the effectiveness of two variants of TFT that may lead to improved effectiveness and/or adherence compared to standard TFT.

Some authors [15, 18, 19] have argued that trauma focused treatment (TFT) is the preferred treatment for patients with CA-PTSD despite earlier mentioned comorbid symptoms in these patients. A recent meta-analysis indeed revealed more symptom improvement after TFT than non-TFT in patients with CA-PTSD [15]. A systematic review also concluded that there is no reason to exclude patients with CA-PTSD from TFT [20]. However, the comorbid symptoms may make it more difficult for those patients to attend weekly treatment sessions, and for therapists to keep the focus on trauma treatment. This has led some researchers to propose that treatment of patients with CA-PTSD may be improved by intensification of TFT. Promising results with an intensified form of TFT in PTSD [21–25] suggest that condensing treatment in a shorter period of time may lead to faster or better treatment results. Reduction of treatment length may not only lead to faster improvement, but also to improved treatment adherence, because there is less time between sessions for anticipatory anxiety to build up [24, 26]. Intensive TFT (up to 18 h of cognitive therapy (CT) delivered in one week) led to faster symptom reduction compared to standard TFT (up to 20 h of weekly CT sessions delivered in 3 months) and equivalent results over 14 weeks [23]. In a veteran population an intensified form of TFT led to faster symptom decline, while it was as effective as regular weekly TFT on the long term [22]. With regard to CA-PTSD, results of a controlled case series design with intensive TFT in adolescents (*N* = 10) also suggest that intensive treatment is safe and acceptable, with an 80% remission rate [24]. Furthermore, results of two open studies in patients with chronic PTSD following multiple traumas, including CA, [21, 25] show that intensive TFT was effective and patient retention high (less than 5% drop-out). Taken together, these studies suggest that intensive TFT (iTFT) may improve overall effectiveness of treatment of CA-PTSD.

Other authors [27–30] have argued that the symptoms and problems frequently observed in patients with CA-PTSD are characteristics of a distinct form of PTSD, referred to as ‘complex PTSD’. Complex PTSD is characterized by prominent emotion regulation difficulties, interpersonal problems and a negative self-concept [30]. The International Society for Traumatic Stress Studies (ISTSS) guidelines recommend ‘phase-based treatment’ as first treatment option for patients with complex PTSD [28]. In phase-based treatment (PBT) the first sessions are focused on addressing emotion regulation and interpersonal problems, which is followed by TFT [31]. This treatment is based on the notion that emotion regulation and interpersonal problems interfere with daily life functioning and that reduction or resolution of these problems can facilitate more effective use of TFT and can best be addressed before starting TFT [31]. PBT has indeed been associated with lower drop-out rates and more complete PTSD remission than supportive treatment followed by TFT [32].

Further research on the treatment of CA-PTSD is needed because of limitations of existing studies. Firstly, no studies have directly compared TFT with PBT or iTFT [15, 18, 33]. Secondly, patients with comorbidities such as dissociation, suicidality and personality disorders have often been excluded from RCTs, limiting the generalizability of the results to the population of CA-PTSD [15, 29, 34, 35]. Thirdly, in most studies participants were predominantly Caucasian and employed, while PTSD is more severe in patients who are unemployed or from minority ethnical backgrounds [15, 29, 36, 37]. Fourthly, many studies have methodological shortcomings such as a lack of blind assessments and no reported data on treatment integrity [15]. Allegiance effects – the unintentional bias due to investigators’ or therapists’ preferences [33, 38] – is a general problem in clinical research. This may be solved by involving researchers with different areas of expertise and allegiances [39].

### Current study

The aim of the current study is to examine the effectiveness of three different treatment strategies for patients with CA-PTSD. We will carry out a randomized controlled trial (RCT) comparing the (cost-)effectiveness and treatment adherence of a well-established form of TFT, prolonged exposure (PE), with two potential improvements of TFT: intensified PE (iPE) and phase-based treatment (PBT). For the iPE group, PE sessions are delivered in 4 weeks (3 sessions per week), PBT consists of Skills Training in Affective and Interpersonal Regulation (STAIR), followed by PE. We expect more PTSD symptom reduction and lower drop-out rates in iPE and PBT than in PE. We also expect that iPE and PBT will be more cost-effective, given that the treatment protocols include fewer (iPE) and shorter (PBT) sessions. We expect that iPE will lead to faster improvement than PE and PBT. Finally, we expect that PBT will be superior to both PE and iPE with respect to improvement in emotion regulation, interpersonal skills and self-esteem. The primary outcome is clinician-rated PTSD symptom severity. Secondary outcomes are loss of PTSD diagnosis, self-reported PTSD symptom severity, treatment adherence, comorbid symptoms severity and cost-effectiveness. Outcomes will be assessed at baseline, after 4, 8 and 16 weeks and at 6 and 12 months follow-up.

### Moderators and mediators

In line with previous work [40], we will investigate whether treatment effects are affected by baseline characteristics such as PTSD symptom severity, comorbid symptoms, emotional maltreatment and avoidance behavior, using between- and within-group moderation tests. We will calculate a ‘personalized advantage index’ (PAI) [41] and trees for treatment-subgroup interactions (QUalitative INteraction Trees; QUINT) to evaluate which pretreatment characteristics are most discriminating in predicting optimal treatment and differential response to treatments with a combination of predictor variables. This may lead to the development of optimal (personalized) treatment sequences [41–43].

As to mediators, moderately strong evidence exists that between-session habitation and change in post-traumatic cognitions mediate the effects of PE, while mixed evidence exists for emotional engagement, inhibition learning and within-session habituation [44]. Mediators of iPE are yet unknown. With regard to PBT, there is some evidence for the mediating effect of both emotion regulation improvement and therapeutic alliance on PBT outcome [31, 45]. More research on mediators is needed, as the number and quality of the studies are limited [44]. In the current study we will examine all above mentioned mediators.

## Methods

### Design

**Fig. 1:**
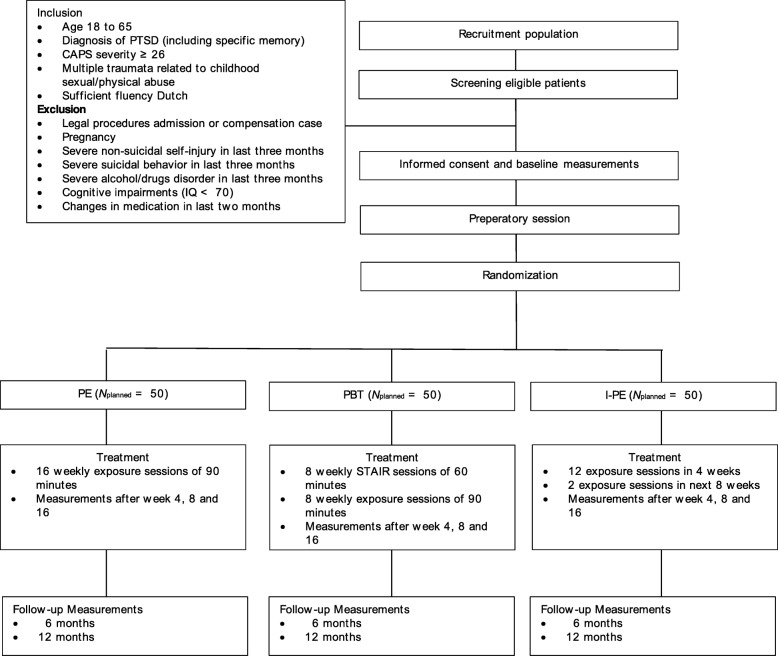
Flowchart of the IMPACT study

The IMPACT study is a multicenter RCT comparing prolonged exposure (PE) with intensified prolonged exposure (iPE) and phase-based treatment (PBT). Participants will be randomly assigned to the conditions. Figure 1 depicts the study flowchart. The research protocol has been approved by the Medical Ethical Committee of Leiden University Medical Center (NL57984.058.16), and is pre-registered at https://clinicaltrials.gov/ct2/show/NCT03194113.

### Recruitment

Participants are recruited at the departments of Psychotrauma of PsyQ Den Haag and PsyQ Rotterdam. Referrals from other treatment centers will also be accepted. After initial screening, potential participants will receive written and oral information about the study. Patients who are interested in participating are invited for the baseline assessment including screening of in- and exclusion criteria and an informed consent procedure. Informed consent will be obtained prior to the assessment.

### Participants

Inclusion criteria of the study are: 1) age 18–65; 2) diagnosis of PTSD as established with the Clinician Administered PTSD Scale (CAPS-5, see instrument section), and at least moderate severity of PTSD-symptoms (CAPS ≥26), and with at least one specific memory for a traumatic event; 3) multiple traumata related to childhood sexual and/or physical abuse that occurred before 18 years of age, committed by a primary caretaker or an authority figure as index event; 4) sufficient fluency in Dutch to complete the treatment and research protocols.

Exclusion criteria are: 1) involvement in a compensation case or legal procedures concerning admission or stay in The Netherlands; 2) pregnancy; 3) severe non-suicidal self-injury (NSSI) which required hospitalization during the past three months; 4) severe suicidal behavior: a suicide attempt during the past three months or acute suicidal ideations with serious intent to die with a specific plan for suicide and preparatory acts; 5) severe disorder in the use of alcohol or drugs in last three months; 6) cognitive impairment (estimated IQ < 70); 7) changes in psychotropic medication in the two months prior to inclusion; and 8) engagement in any current psychological treatment.

### Sample size

Our sample size calculations are based on the intention to detect at least moderate effect size differences (d = .40) among conditions. To detect this effect size difference in PTSD severity with alpha = .05 (2-tailed) and a power of 0.8, 50 participants per condition are needed. We expect some drop-out which will result in a lower power due to missing values. However, we calculated the sample size based on the conservative assumption that the correlation between the baseline and all further post measurements is 0 and the correlation between post measurements is 1, since we do not have a good estimation for the correlation between the outcome measurements yet. Thus, the actual power is expected to be considerably higher than 0.8 due to the multiple measurement design correcting for power loss due to drop-out [46, 47].

### Procedure

Before randomization, patients complete a baseline assessment of the study. In the preparatory session, patients receive detailed information about the treatment and research procedures and about practical considerations, such as availability. Randomization is carried out by an independent researcher from Leiden University who uses a computerized randomization sequence of permutated blocks of six patients, stratified by gender. Patients are regarded as treatment drop-out if they stop therapy prematurely and as measurement drop-out if they refuse or do not show up for follow-up measurements. Early responders are defined by a score below 16 on the PTSD checklist for DSM-5 (PCL-5) for three consecutive weeks with agreement between patient, therapist and supervisor about finishing the therapy early [48, 49]. Measurements will take place at baseline, during the therapy (after 4 weeks, 8 weeks and 16 weeks) and follow-up measurements after 6 and 12 months. All measurements are performed by trained and supervised interviewers, who are blind to treatment condition. Patients and their therapists also fill out self-report questionnaires before therapy sessions and fill out questionnaires about harm expectancies and distress during the exposure therapy.

### Therapists and training

Before participation in the trial, master’s level therapists attend a two-day training in prolonged exposure and a two-day training in STAIR. At the end of these trainings, the therapists have to pass an exam with pilot patients, which is graded by the supervisors of the study. During the study, all therapists receive weekly supervision in (i)PE (by AM and RK) and PBT (by MC and IW). All treatment locations offer the three types of treatment and all therapists receive the same amount of supervision and training. Adherence to the treatment protocols will be checked by independent observers, who will rate randomly selected videotaped therapy sessions.

### Prolonged exposure therapy

Prolonged exposure therapy (PE) is delivered in 16 weekly sessions of 90 min. The treatment manual is based on the PE protocol by Foa, Hembree, & Rothbaum (2007) [50].

Treatment sessions consist of imaginal exposure (repeated recounting of the most anxiety provoking traumatic memories and processing related thoughts and feelings), and exposure in vivo (approaching trauma-related situations). Between sessions, participants listen to audio recordings of the imaginal exposure on a daily basis, and complete in-vivo homework assignments.

### Intensified prolonged exposure therapy

Intensified prolonged exposure therapy (iPE) involves three weekly sessions of 90 min PE for a period of four weeks (12 sessions total), followed by two PE sessions after one and two months (14 sessions total). The same protocol is used as in the PE condition with some minor changes for practical considerations. For instance, when two treatment sessions are given on consecutive days patients are instructed to do combined homework of both sessions. After the first 12 sessions, patients are instructed to keep doing imaginal exposure and exposure in vivo homework for the 13th and 14th sessions. For practical considerations, two therapists deliver the iPE sessions alternately.

### Phase-based therapy

Phase-based therapy (PBT) is delivered in 8 weekly 60 min STAIR sessions [51], followed by 8 weekly 90 min PE sessions. STAIR is a manualized skills training, adapted from dialectical behavior therapy and cognitive behavioral therapy [52]. The first four STAIR sessions focus on improving emotion regulation skills, including labeling and identifying feelings, emotion management, distress tolerance and the acceptance of feelings and experiencing positive emotions. The last four STAIR sessions focus on developing interpersonal skills and address exploration and revision of maladaptive schemas, the conflict between trauma generated feelings and interpersonal goals in the present, differences in power and control and flexibility in interpersonal situations with differences in power [31]. Throughout the treatment, patients receive psychoeducation, especially about the connection between the traumatic events during their childhood and the effect it has on their present thoughts, feelings and behavior. After these eight sessions the protocol continues with the standard PE protocol [50]. This differs from the standard STAIR protocol, which continues with the Narrative Story Telling (NST) protocol [53].

### Instruments

In Table 1, an overview is presented of all the included measures and measurement points.

**Table 1:** Overview of the measurements per time point

Clinical interview	Construct	T0	T1	T2	T3	T4	T5
MINI	Axis-1 disorders	X			X	X	X
CAPS-5	PTSD	X	X	X	X	X	X
CPI	Complex PTSD	X	X	X	X	X	X
SCID II	Personality disorders	X				X	
DSP-I	Dissociation	X	X	X	X	X	X
Self-report							
Demographics	Demographics	X					
LEC-5	Traumata	X					
CTQ	Childhood maltreatment	X				X	
PCL-5^b^	PTSD symptoms	X	X	X	X	X	X
DERS^b^	Emotion regulation	X	X	X	X	X	X
ICD-11	Complex PTSD	X	X	X	X	X	X
BDI-II	Depression	X	X	X	X	X	X
PTCI	Posttraumatic cognitions	X	X	X	X	X	X
DES	Dissociation	X	X	X	X	X	X
SDQ-5	Somatoform Dissociation Questionnaire	X	X	X	X	X	X
DERS	Emotion regulation	X	X	X	X	X	X
TIC-P	Direct/indirect costs	X			X	X	X
IIP	Interpersonal problems	X	X	X	X	X	X
MOS	Social support	X	X	X	X	X	X
RSES	Self-esteem	X	X	X	X	X	X
ZAV	Anger	X	X	X	X	X	X
ACS	Attentional control	X	X	X	X	X	X
LEIDS	Cognitive reactivity				X		
Treatment credibility	Treatment credibility	X			X		
Treatment Goals	Treatment goals	X					
EQ-5L5D	Quality of life	X	X	X	X	X	X
WAI^a^	Working alliance						
Cognitive task							
Avoidance task	Avoidance behavior	X					
Process variables		Measurement moment					
HE	Harm expectancies	Prior and after (imaginal) exposure					
SUD	Subjective distress	Multiple times during (imaginal) exposure					

*MINI* Mini-international Neuropsychiatric Interview, *CAPS-5* Clinician Adminstered PTSD Scale, *CPI* Complex PTSD Items, *SCID II* Structured Clinical Interview for DSM-IV axis-II personality disorders, *DSP-I* Dissociatief Subtype van PTSS, *LEC-5* Life Events Checklist for DSM-5, *CTQ* Childhood Trauma Questionnaire, *PCL-5* PTSD Checklist for DSM-5, *DERS* Difficulties in Emotion Regulation Scale, *ICD-11* International Classiciation of Diseases-11, *BDI-II* Beck Depression Inventory-II, *PTCI* The posttraumatic cognitions inventory, *DES* Dissociative Experiences Scales, *SDQ-5* Somatoform Dissociation Questionnaire-5, *DERS* Difficulties in Emotion Regulation Scale; *TIC-P* Trimbos and iMTA questionnaire on Costs associated with Psychiatric illness, *IIP* Inventory of Interpersonal Problems, *MOS* Medical Outcomes Study, *RSES* Rosenberg Self-Esteem Scale, *ZAV* Zelf Analyse Vragenlijst, *ACS* Attentional Control Scale, *LEIDS* The Leiden Index of Depression Sensitivity, *EQ-5D-5 L* EuroQoL 5 Dimensions 5 Levels, *WAI* Working Alliance Inventory T0 = baseline, T1 = 4 weeks, T2 = 8 weeks, T3 = 16 weeks, T4 = 26 weeks, T5 = 52 weeks

^a^WAI is self-administered by the patient and therapist 4 times during the course of treatment before the start of the treatment sessions

^b^PCL-5 and DERS are self-administered weekly before the therapy session by the patient

#### Clinician-rated PTSD symptom severity

PTSD diagnosis and symptom severity are assessed with the Clinical Administered PTSD scale (CAPS-5) [54]. The CAPS-5 has recently been validated for the DSM-5 diagnosis of PTSD and has been translated into Dutch [55]. The CAPS-5 has good correspondence with CAPS-4 (*kappa* = .83) for the diagnosis of PTSD and a high internal consistency (α = .88) and test-retest reliability (*ICC* = .78) for the total severity score [56]. Response to the treatment is defined as an improvement of at least 6 points on the CAPS-5 [57]. Remission is defined as response to treatment, loss of diagnosis and a symptom severity score below 26.

#### Self-reported PTSD symptom severity

Posttraumatic symptom severity is also measured with the PTSD checklist for DSM-5 (PCL-5). The PCL-5 has a high internal consistency (*a* = .94) and test-retest reliability (*r* = .82) [58, 59].

#### Comorbid symptom severity

To measure clinician-rated symptoms that have been proposed to define complex PTSD [28] we use three clinical administered items measuring problems with emotion regulation, interpersonal difficulties and low self-esteem (Complex PTSD items, CPI). Emotion regulation, interpersonal difficulties and self-esteem are also assessed with the Trauma Questionnaire of the International Classification of Diseases, 11th edition (ICD-11) [60]. Additionally, emotion regulation difficulties are measured with the Difficulties in Emotion Regulation Scale (DERS) [61]. Interpersonal problems are measured with the Inventory of Interpersonal Problems (IIP-32) [62, 63] and self-esteem with the Rosenberg Self-Esteem Scale (RSES) [64]. Clinician-rated dissociative symptom severity is measured with the two items about the dissociative subtype of PTSD in the CAPS-5. Also, we will also use a new clinical interview for the Dissociative Subtype in PTSD (DSP-I) [65]. Self-reported dissociative symptom severity is measured with the the Dissociative Experiences Scale (DES) [66] and the Somatoform Dissociation Questionnaire (SDQ) [67].

Comorbid axis-1 disorders (DSM-IV) are measured with the Mini International Neuropsychiatric Interview (MINI) [68]. Depression severity is measured with the Beck Depression Inventory, 2nd edition (BDI-II-NL) [69]. Cognitive reactivity and specifically suicidal reactivity is assessed with the Leiden Index of Depression Sensitivity (LEIDS) [70].

Personality disorders are measured with the Structured Clinical Interview for DSM-IV Personality Disorders (SCID-II) [71].

Moreover, anger, negative cognitions, social support and attentional control are measured using self-report questionnaires State-Trait Anger Scale (ZAV) [72], the Posttraumatic Cognitions Inventory (PTCI) [73, 74] the MOS [75, 76] and the Attentional Control Scale (ACS) [76].

#### Trauma history

The LEC-5 [77, 78] measures any experienced traumatic event and the CTQ (Childhood Trauma Questionnaire) will be used to measure childhood trauma specifically [79, 80].

#### Treatment variables

Prior, during and immediately after (imaginal) exposure, Subjective Units of Distress (SUD) ratings are assessed and prior and after exposure harm expectancies are assessed. Treatment credibility of the three therapies will be checked with the adapted Treatment Credibility Scale [81]. Additionally, the Working Alliance Inventory (WAI) [82–84] will be used to examine therapeutic alliance. The treatment goals of the patients are assessed with an adapted version of the Bern inventory of treatment goals [85].

#### Cost-effectiveness

Quality of Life is measured with the EQ-5D-5 L [86, 87]. The EQ-5D-5 L questionnaire will also be used as cost-effectiveness measurement with the use of the social tariffs of the EuroQol.

Moreover, cost-effectiveness is determined with the Trimbos/iMTA questionnaire for costs associated with Psychiatric Illness (TiC-P) [88] which measures the (in)direct costs of illness (health care use and lost productivity), and is specifically developed for the Dutch Healthcare system.

#### Avoidance task

A classical associative learning paradigm is administered to measure avoidance behaviors. In this task, emotional, anxiety provoking pictures from the International Affective Picture System (IAPS)- set are used as unconditioned stimulus (US), and pictures of an office containing a light, that changes color (blue, red, yellow) as the conditioned stimulus (CS). Participants can avoid the US by pressing a button, but success is dependent on the CS [89].

### Analyses

Data analyses will be based on intention-to-treat analyses. All randomized patients will be included in the analyses. Due to the structured data, we will use multiple imputation of multilevel data which takes the levels within the data into account [90].

Primary and secondary continuous outcome parameters will be analyzed with multilevel mixed models using a repeated measurement design to correct for the dependencies among the observations [91, 92]. Dichotomous secondary outcome parameters will be analyzed with multilevel logistic regression. The intraclass correlation will be determined to give an indication about these dependencies and determine the residuals which can be explained within and between patients [92]. The models will be fitted with the lme4 package in R and with a FML estimation method [93]. The models will be nested, so the models are compared with the likelihood ratio test (LRT) [94]. All assumptions of the models will be checked to ensure the reliability of the results. When major assumptions are violated, clustered bootstrap will be used, since this method can handle structured data and has less stringent assumptions than multilevel models. Cost-utility analysis will be based on patient reports (societal costs per QALY), and cost-calculator spreadsheet model (BIA). The economic evaluation will also be based on analysis to treat; standard Dutch unit prices will be used.

For moderation and mediation analyses, regression based approaches will be used with the PROCESS macro in SPSS [95]. For moderation analyses with multiple time points, linear mixed models will be used with an interaction effect between time and the moderation variable of interest. For between treatment moderation analyses the three-way interaction between the moderator, treatments and time will be calculated. For calculation of the personalized advantage index we will use leave-one-out cross validation to generate the counterfactual prediction per patient using prognostic and prescriptive variables from moderation analyses and generate the PAI, the magnitude of the predicted difference of receiving the predicted optimal treatment versus the non-optimal treatment [41, 96]. For the Trees for treatment-subgroup interactions we will use the R-package quint which uses a stepwise tree building algorithm to detect treatment by subgroup interaction allowing all possible predictor combinations in the model. The algorithm subdivides all patients in terminal nodes based on their patient characteristics and further assigns patients to nodes in which either one of the treatment is better than the other or both treatments are equally effective [42, 43].

## Discussion

Completion of this RCT will provide more knowledge about the relative effectiveness of three treatment strategies for CA-PTSD. We will directly compare the effects of a well-established treatment (prolonged exposure) and two treatment innovations (intensified prolonged exposure and phase-based treatment) in this difficult to treat patient population. Furthermore, cost-effectiveness of the three interventions will be examined. Finally, moderation and mediation analyses will provide more information for whom and under which conditions these treatments are most effective. Ultimately, this might assist clinicians in personalizing treatment indications and optimizing treatment delivery.

### Methodological considerations

We expect to include a cultural and socioeconomic diverse sample, since the participating centers are located in large cities. We protect the generalizability of the findings by using few exclusion criteria. The relatively long follow-up measurements of 6 and 12 months will provide insights in the long-term effects of the therapies. Every type of treatment is supervised by expert supervisors of that specific method. Additionally, all therapists are trained and supervised in both PE and PBT. This prevents biases to the internal reliability of the study and is essential for a meaningful interpretation of the results [39].

Limitations of this study are that not all eligible patients will agree to participate in the study which could result in selection bias. Especially the iPE condition could lead to selection bias since it is more demanding in terms of time investment in the first weeks of the treatment. All reasons of patients to decline participation in the study will be carefully monitored to ensure the generalizability of the results and for implementation purposes. Another limitation is that patients have one therapist in PE and PBT, but two alternating therapists in the iPE condition. This may influence the therapeutic alliance and consequently the results of the treatment. We will assess whether therapeutic alliance indeed differs between condition and, if so, whether this has any influence on treatment results.

## Conclusion

Patients with CA-PTSD have a high burden of disease. Currently, there is no consensus on treatment-guidelines for this patient group. The results of this study may have important implications for the treatment of patients with CA-PTSD.

## Abbreviations

CAPS-5: Clinician Administered PTSD Scale
CA-PTSD: Childhood Abuse related Posttraumatic Stress Disorder
CT: Cognitive Therapy
iPE: Intensified form of PE
ISTSS: The International Society for Traumatic Stress Studies
iTFT: Intensified form of Trauma Focused Treatment
PAI: Personalized Advantage Index
PBT: Phase-Based Treatment
PCL-5: PTSD Checklist for DSM-5
PE: Prolonged Exposure
PTSD: Posttraumatic Stress Disorder
RCT: Randomized Controlled Trial
STAIR: Skills Training in Affective and Interpersonal Regulation
TFT: Trauma Focused Treatment
